# Supplementary material for: Post-competition recovery strategies in elite male soccer players. Effects on performance: A systematic review and meta-analysis
Source: PLoS One. 2020 Oct 2;15(10):e0240135. doi: 10.1371/journal.pone.0240135 (PMC7531804; doi:10.1371/journal.pone.0240135)
Supplement: S2 File — (PDF) [file pone.0240135.s002.pdf]

II– Search strategies

All databases were searched using the Boolean method with the following terms (1 AND 2 AND 3 AND 4 AND 5 AND 6 AND 7):

1. Athletes: athletes OR players OR participants
2. Sport: sport OR team sport (any of them) OR exercise OR football OR soccer
3. Recovery: recovery OR recovery strategies OR recovery modalities OR recovery methods
4. Match: match OR game OR competition OR post-match OR post-game OR post-competition
5. Recovery strategies: cold water immersion OR active recovery OR passive recovery OR stretching OR stretch OR compression garments OR massage OR whole-body vibration OR electrical stimulation OR foam roller OR foam rolling OR contrast baths OR chamber OR cooling.
6. Performance: performance OR muscle soreness OR muscle damage OR delayed onset muscle soreness OR biochemical markers OR heart rate OR tendon damage OR countermovement jump OR countermovement jump OR sprint OR agility OR creatine kinase OR cortisol OR testosterone
7. Feeling perception: perception OR perceived OR rate perceived exertion OR wellness OR sleep OR total quality recovery

Medline (PubMed)

(athletes OR players OR participants) AND (sport OR team sport OR exercise OR football OR soccer) AND (recovery OR recovery strategies OR recovery modalities OR recovery methods) AND (match OR game OR competition OR post-competition OR post-match) AND (cold water immersion OR active recovery OR passive recovery OR stretching OR stretch OR compression garments OR massage OR whole-body vibration OR electrical stimulation OR foam roller OR foam rolling OR contrast baths OR chamber OR cooling) AND (performance OR muscle soreness OR muscle damage OR doms OR delayed onset muscle soreness OR biochemical markers OR heart rate OR tendon damage OR countermovement jump OR Counter movement jump OR sprint OR agility OR creatine kinase OR cortisol OR testosterone) AND (perception OR perception feeling OR perceived OR rate perceived exertion OR wellness OR sleep OR total quality recovery)

SCOPUS

(((ALL (*"recovery"*)) OR (ALL (*"recovery strategies"*)) OR (ALL (*"recovery modalities"*)) OR (ALL (*"recovery methods"*))) AND ((ALL (*"match"*)) OR (ALL (*"game"*)) OR (ALL (*"competition"*)) OR (ALL (*"post-match"*)) OR (ALL (*"post-competition"*)) OR (ALL (*"post-game"*))) AND ((((ALL (*"cold water immersion"*)) OR (ALL (*"active recovery"*)) OR (ALL (*"passive recovery"*)) OR (ALL (*"stretch"*)) OR (ALL (*"stretching"*)) OR (ALL (*"compression garments"*)) OR (ALL (*"massage"*))) OR (ALL (*"whole body vibration"*)) OR (ALL (*"electrical stimulation"*)) OR (ALL (*"foam roller"*)) OR (ALL (*"foam rolling"*)) OR (ALL (*"contrast baths"*)) OR (ALL (*"chamber"*))) OR (ALL (*"cooling"*))) AND ((((ALL (*"performance"*)) OR (ALL (*"muscle soreness"*)) OR (ALL (*"muscle damage"*)) OR (ALL (*"delayed onset muscle soreness"*)) OR (ALL (*"biochemical markers"*)) OR (ALL (*"heart rate"*)) OR (ALL (*"tendon damage"*))) OR (ALL (*"countermovement jump"*)) OR (ALL (*"sprint"*)) OR (ALL (*"counter movement jump"*)) OR (ALL (*"agility"*)) OR (ALL (*"creatine kinase"*)) OR (ALL (*"cortisol"*))) OR (ALL (*"testosterone"*))) AND ((ALL (*"perception"*)) OR (ALL (*"perceived"*)) OR (ALL (*"rate perceived exertion"*)) OR (ALL (*"wellness"*)) OR (ALL (*"sleep"*)) OR (ALL (*"total quality recovery"*)))) AND ((ALL (*"athletes"*)) OR (ALL (*"players"*)) OR (ALL (*"participants"*))) AND ((ALL (*"sport"*)) OR (ALL (*"team sport"*)) OR (ALL (*"exercise"*)) OR (ALL (*"football"*)) OR (ALL (*"soccer"*)))) AND (LIMIT-TO (DOCTYPE , *"ar"*)) AND (LIMIT-TO (LANGUAGE , *"English"*) OR LIMIT-TO (LANGUAGE , *"Spanish"*))

SPORTDiscus

((athletes) OR (players) OR (participants)) AND ((sport) OR (team sport) OR (exercise) OR (football) OR (soccer)) AND ((recovery) OR (recovery strategies) OR (recovery modalities) OR (recovery methods)) AND ((match) OR (game) OR (competition) OR (post-match) OR (post-game) OR (post-competition)) AND ((cold water immersion) OR (active recovery) OR (passive recovery) OR (stretching) OR (stretch) OR (compression garments) OR (massage) OR (whole-body vibration) OR (electrical stimulation) OR (foam roller) OR (foam rolling) OR (contrast baths) OR (chamber) OR (cooling)) AND ((performance) OR (muscle soreness) OR (muscle damage) OR (delayed onset muscle soreness) OR (doms) OR (biochemical markers) OR (heart rate) OR (tendon damage) OR (countermovement jump) OR (counter movement jump) OR (sprint) OR (agility) OR (creatine kinase) OR (cortisol) OR (testosterone)) AND ((perception) OR (perceived) OR (rate perceived exertion) OR (wellness) OR (sleep) OR (total quality recovery))

ISI WEB OF SCIENCE (WOS)

(TS=("athletes") OR TS=("players") OR TS=("participants")) AND (TS=("sport") OR TS=("team sport") OR TS=("exercise") OR TS=("football") OR TS=(“soccer”)) AND (TS=(recovery) OR TS=("recovery strategies") OR TS=("recovery modalities") OR TS=("recovery methods")) AND (TS=("match") OR TS=("game") OR TS=("competition") OR TS=("post-match") OR TS=("post-game") OR TS=("post-competition") AND (TS=("cold water immersion ") OR TS=("active recovery") OR TS=("passive recovery") OR TS=("stretching") OR TS=("stretch") OR TS=("compression garments ") OR TS=("massage") OR TS=("whole-body vibration") OR TS=("electrical stimulation") OR TS=("foam roller") OR TS=("foam rolling") OR TS=("contrast baths") OR TS=("chamber") OR TS=(“cooling”)) AND (TS="performance") OR TS=("muscle soreness") OR TS=("delayed onset muscle soreness") OR TS=("biochemical markers ") OR TS=("heart rate") OR TS=("tendon damage") OR TS=("countermovement jump") OR TS=("counter movement jump") OR TS=("sprint") OR TS=("agility") OR TS=("creatine kinase") OR TS=("cortisol") OR TS=("testosterone")) AND (TS=("perception") OR TS=("perceived") OR TS=("rate perceived exertion") OR TS=("wellness") OR TS=("sleep") OR TS=("total quality recovery"))

CINAHL

(athletes OR players OR participants) AND (sport OR team sport OR exercise OR football OR soccer) AND (recovery OR recovery strategies OR recovery modalities OR recovery methods) AND (match OR game OR competition OR post-competition OR post-match) AND (cold water immersion OR active recovery OR passive recovery OR stretching OR stretch OR compression garments OR massage OR whole-body vibration OR electrical stimulation OR foam roller OR foam rolling OR contrast baths OR chamber OR cooling) AND (performance OR muscle soreness OR muscle damage OR doms OR delayed onset muscle soreness OR biochemical markers OR heart rate OR tendon damage OR countermovement jump OR Counter movement jump OR sprint OR agility OR creatine kinase OR cortisol OR testosterone) AND (perception OR perception feeling OR perceived OR rate perceived exertion OR wellness OR sleep OR total quality recovery)

Cochrane Central Register of Controlled Trials

(athletes OR players OR participants) AND (sport OR team sport OR exercise OR football OR soccer) AND (recovery OR recovery strategies OR recovery modalities OR recovery methods) AND (match OR game OR competition OR post-competition OR post-match) AND (cold water immersion OR active recovery OR passive recovery OR stretching OR stretch OR compression garments OR massage OR whole-body vibration OR electrical stimulation OR foam roller OR foam rolling OR contrast baths OR chamber OR cooling) AND (performance OR muscle soreness OR muscle damage OR doms OR delayed onset muscle soreness OR biochemical markers OR heart rate OR tendon damage OR countermovement jump OR Counter movement jump OR sprint OR agility OR creatine kinase OR cortisol OR testosterone) AND (perception OR perception feeling OR perceived OR rate perceived exertion OR wellness OR sleep OR total quality recovery)
