# Supplementary material for: Post-competition recovery strategies in elite male soccer players. Effects on performance: A systematic review and meta-analysis
Source: PLoS One. 2020 Oct 2;15(10):e0240135. doi: 10.1371/journal.pone.0240135 (PMC7531804; doi:10.1371/journal.pone.0240135)
Supplement: S3 File — (PDF) [file pone.0240135.s003.pdf]

III-Data extraction form for randomised controlled trials

Reviewer:	Date:	Study number:

GENERAL STUDY INFORMATION

First author (e.g. Smith F):

Correspondence to: ☐not provided

Title:

Journal:

Year of publication: **Vol.:** **Num.:** **Pages:**

Country: **Language:**

Sources of support:

STUDY DESIGN

☐ Randomised Controlled Trial

☐ Cluster Randomised Controlled Trial

Details:

Setting: ☐Unicenter ☐Multicenter (☐National / ☐International) ☐ Detail not provided

Recruitment period (months): ☐ Detail not provided

STUDY POPULATION AND PARTICIPANTS

Study population description:

Inclusion criteria:

Exclusion criteria:

Flow of participants		
	**Groups Intervention / Control**	**Reasons/Details**
**Invited to participate and/or screened for eligibility**		
**Declined to participate**		
**Excluded**		
**Randomized**		
**Dropouts**		
**Completed**		
**Analysed**		

Baseline characteristics				
	**Total: (n=)**	**Intervention (n=)**	**Control (n=)**	**Between group difference (statistically significant)**
**Age**				☐ Yes ☐ No
**Gender**				☐ Yes ☐ No
**Weight**				☐ Yes ☐ No
**Height**				☐ Yes ☐ No
**BMI**				☐ Yes ☐ No
				☐ Yes ☐ No

INTERVENTION

Intervention general description and objectives:

Duration (weeks/months):

Intervention characteristics					
	**Recovery method**	**Type & Brand**	**Duration (sets & reps)**	**Frequency**	**Attendance / compliance rate**
**Intervention:**					
**Control:**					

DATA COLLECTION

Data collection procedures: PRIMARY OUTCOME			
**Variables**	**Responsible** (who collected data**)**	**Method** (scale, instrument,etc.)	**Details**
**Physical performance**			

Data collection procedures: SECONDARY OUTCOMES			
**Variables**	**Responsible** (who collected data**)**	**Method** (scale, instrument,etc.)	**Details**
**Other**			
**Subjective perception**			
**Technical & Tactical**			
**Physiological**			

RESULTS

Drop-outs		
**Group**	**Num. (%)**	**Description/Reasons**
**Intervention**		
**Control**		

Results				
Intervention (n=)				
**Variable (and timing)**	**Baseline**	**Post-competition**	**P-value**	**Effect size**

Results
**Control (n=)**

Variable and timing	Baseline	Post-competition	P-value	Effect size

ADVERSE EVENTS

Adverse events collection and reporting			
**Registering adverse events**	☐ Yes ☐ No		
**Results**	**Total:**	**Intervention group:**	**Control group:**
**Other descriptions:**			

CONCLUSIONS

Conclusions
Primary outcome:

COMMENTS
(Add general comments if relevant)

METHODS

Methodological details		
		Description / details
**Eligibility criteria specified**	☐ Yes ☐ No	
**Power calculation**	☐ Yes ☐ No	
**Method of randomization**	☐ Adequate / computer generated<br>☐ Inadequate<br>☐ Not reported	
**Allocation concealment**	☐ Adequate<br>☐ Doubtful<br>☐ Inadequate<br>☐ Not reported	
**Blinding**	Participants: ☐ Yes ☐ No<br>Coach/es: ☐ Yes ☐ No<br>Outcome assessor/s: ☐Yes ☐ No ☐ Not reported	
**Handling of withdrawals description**	☐ Yes ☐ No	
**Pre-published study protocol**	☐ Yes ☐ No	

APPENDIX 2:

RISK OF BIAS ASSESSMENT FORM
FOR RANDOMISED CONTROLLED TRIALS

Reviewer:

First author (year): ( ) Assessment data: Study number:		
**Bias domain**	**Author's judgment (low, unclear, high)**	**Support for judgment**
Random sequence generation (selection bias)		
Allocation concealment (selection bias)		
Blinding of participants and researchers (performance bias)		
Blinding of outcome assessment (detection bias)		
Incomplete outcome data (attrition bias)		
Selective reporting (reporting bias)		
Other bias		
