# Supplementary material for: Post-competition recovery strategies in elite male soccer players. Effects on performance: A systematic review and meta-analysis
Source: PLoS One. 2020 Oct 2;15(10):e0240135. doi: 10.1371/journal.pone.0240135 (PMC7531804; doi:10.1371/journal.pone.0240135)
Supplement: S1 Table — SMD: Standardized Mean Difference; CMJ: counter movement jump; MVC: maximal voluntary contraction; DOMS: delayed onset muscle soreness; CK: creatine kinase; CRP: C-reactive protein; QS: quadriceps; HS: hamstrings; CS: calf. aAscensão evaluated at 24-hours and 48-hours, while Fullagar evaluated at 20-hours and 44-hours post-match. (DOCX) [file pone.0240135.s004.docx]

| **Variable** | | **Study** | | **Mean Difference**  **[95% CI]** | | **Random effects model** | | **P-value** | **SMD**  **[95% CI]** | | |
| --- | --- | --- | --- | --- | --- | --- | --- | --- | --- | --- | --- |
| **Primary outcomes** | CMJ  24h | Ascensão et al. 2011 | | -6.70 [-14.00; 0.60] | | -1.06 [-2.87; 1.36] | | 0.391 | -0.29 [-0.72; 0.18] | | |
|  |  | Marqués-Jiménez (a) et al. 2018 | | -0.21 [-3.35; 2.92] | |  |  |  |  |  |  |
|  |  | Clifford et al. 2018 | | -0.60 [-3.31; 2.11] | |  |  |  |  |  |  |
|  | CMJ  48 h | Ascensão et al. 2011 | | -3.70 [-9.20; 1.80] | | 0.10 [-1.76; 1.97] | | 0.914 | -0.05 [-0.49; 0.41] | | |
|  |  | Marqués-Jiménez (a) et al. 2018 | | 0.56 [-3.15; 4.28] | |  |  |  |  |  |  |
|  |  | Clifford et al. 2018 | | 0.60 [-1.60; 2.82] | |  |  |  |  |  |  |
|  | 20-m sprint  24 h | Ascensão et al. 2011 | | 0.16 [0.10; 0.31] | | 0.08 [-0.02; 0.199] | | 0.142 | 0.53 [-0.09; 1.15] | | |
|  |  | Marqués-Jiménez (a) et al. 2018 | | 0.04 [-0.05; 0.13] | |  |  |  |  |  |  |
|  | 20-m sprint  48 h | Ascensão et al. 2011 | | 0.31 [0.09; 0.52] | | 0.14 [-0.16; 0.44] | | 0.412 | 0.34 [-0.85; 1.54] | | |
|  |  | Marqués-Jiménez (a) et al. 2018 | | -0.01 [-0.08; 0.08] | |  |  |  |  |  |  |
|  | MVC  24 h | Ascensão et al. 2011 | | -8.00 [-157.90; 141.90] | | -60.37 [-169.03; 48.29] | | 0.276 | -1.06 [-2.25; 0.13] | | |
|  |  | Clifford et al. 2018 | | -118.36 [-276.10; 39.38] | |  |  |  |  |  |  |
|  | MVC  48 h | Ascensão et al. 2011 | | -146.00 [-270.38; -21.61] | | -99.54 [-210.44; 11.35] | | 0.078 | -0.72 [-1.57; 0.13] | | |
|  |  | Clifford et al. 2018 | | -30.63 [-190.09; 128.83] | |  |  |  |  |  |  |
| **Secondary outcomes** | QS DOMS Post-match  24 h | | Ascensão et al. 2011 | | 0.33 [-1.11; 1.77] | -0.17 [-1.37; 1.04] | 0.731 | | | -0.15 [-0.72; 0.31] |  |
|  |  |  | Marqués-Jiménez (b) et al. 2018 | | -1.30 [-3.48; 0.88] |  |  |  |  |  |  |
|  | QS DOMS Post-match  48 h | | Ascensão et al. 2011 | | -0.20 [-1.45; 1.05] | -1.61 [-4.60; 1.39] | 0.293 | | | -0.61 [-1.45; 0.22] |  |
|  |  |  | Marqués-Jiménez (b) et al. 2018 | | -3.27 [-3.90; 0.08] |  |  |  |  |  |  |
|  | HS DOMS Post-match  24 h | | Ascensão et al. 2011 | | 0.31 [-1.56; 2.18] | 0.03 [-1.52; 1.58] | 0.972 | | | -0.42 [-0.95; 0.11] |  |
|  |  |  | Marqués-Jiménez (b) et al. 2018 | | -0.58 [-3.32; 2.16] |  |  |  |  |  |  |
|  | HS DOMS Post-match  48 h | | Ascensão et al. 2011 | | -0.35 [-2.11; -1.40] | -1.01 [-2.72; 0.71] | 0.250 | | | -0.04 [-0.57; 0.48] |  |
|  |  |  | Marqués-Jiménez (b) et al. 2018 | | -2.17 [-4.75; 0.411] |  |  |  |  |  |  |
|  | CS DOMS Post-match  24 h | | Ascensão et al. 2011 | | -0.76 [-2.30; 0.78] | -1.08 [-2.42; 0.25] | 0.111 | | | -0.49 [-1.01; 0.06] |  |
|  |  |  | Marqués-Jiménez (b) et al. 2018 | | -2.70 [-4.75; 0.61] |  |  |  |  |  |  |
|  | CS DOMS Post-match  48 h | | Ascensão et al. 2011 | | -0.47 [-1.88; 0.94] | -1.09 [-2.09; -0.09] | 0.009 | | | -0.47 [-1.01; -0.06] |  |
|  |  |  | Marqués-Jiménez (b) et al. 2018 | | -2.46 [-5.23; 0.32] |  |  |  |  |  |  |
|  | CK  24 h^a^ | | Ascensão et al. 2011 | | 586.00 [541.97; 630.02] | 586.48 [542.53; 630.42] | <0.0001 | | | 0.88 [0.33; 1.43] |  |
|  |  |  | Fullagar et al. 2016 | | 719.00 [-13.27; 1451.27] |  |  |  |  |  |  |
|  | CK  48 h^a^ | | Ascensão et al. 2011 | | 654.00 [613.69; 694.30] | 653.23 [613.00; 693.46] | <0.0001 | | | 0.86 [0.03; 1.70] |  |
|  |  |  | Fullagar et al. 2016 | | 449.00 [-209.03; 1107.03] |  |  |  |  |  |  |
|  | CRP  24 h^a^ | | Ascensão et al. 2011 | | 1.35 [1.07; 1.62] | 1.32 [1.055; 1.560] | <0.0001 | | | 1.41 [0.01; 2.82] |  |
|  |  |  | Fullagar et al. 2016 | | 0.9 [-0.24; 2.04] |  |  |  |  |  |  |
|  | CRP  48 h^a^ | | Ascensão et al. 2011 | | -0.15 [-0.46; 0.16] | -0.12 [-0.848; 1.448] | 0.433 | | | -0.02 [-0.54; 0.50] |  |
|  |  |  | Fullagar et al. 2016 | | 0.3 [-0.85; 1.45] |  |  |  |  |  |  |
