# Supplementary material for: Post-competition recovery strategies in elite male soccer players. Effects on performance: A systematic review and meta-analysis
Source: PLoS One. 2020 Oct 2;15(10):e0240135. doi: 10.1371/journal.pone.0240135 (PMC7531804; doi:10.1371/journal.pone.0240135)
Supplement: S2 Table — SMD: Standardized Mean Difference; CMJ: counter movement jump; MVC: maximal voluntary contraction; DOMS: delayed onset muscle soreness; CK: creatine kinase; CRP: C-reactive protein; QS: quadriceps; HS: hamstrings; CS: calf. aAscensão evaluated at 24-hours and 48-hours, while Fullagar evaluated at 20-hours and 44-hours post-match. (DOCX) [file pone.0240135.s005.docx]

| **Variable** | | **Study** | | **Mean Difference**  **[95% CI]** | | **Random effects model** | | **P-value** | **SMD**  **[95% CI]** | | |
| --- | --- | --- | --- | --- | --- | --- | --- | --- | --- | --- | --- |
| **Primary outcomes** | CMJ basal  24h | Ascensão et al. 2011 | | -11.11 [-19.03; -3.16] | | -3.31 [-7.70; 1.07] | | 0.1383 | -0.78 [-1.63; 0.07] | | |
|  |  | Marqués-Jiménez (a) et al. 2018 | | -1.25 [-5.77; 3.27] | |  |  |  |  |  |  |
|  |  | Clifford et al. 2018 | | -1.52 [-4.36; 1.32] | |  |  |  |  |  |  |
|  | CMJ basal  48 h | Ascensão et al. 2011 | | -9.60 [-16.80; -2.39] | | -3.40 [-7.02; 0.21] | | 0.0655 | -0.66 [-1.51; 0.19] | | |
|  |  | Marqués-Jiménez (a) et al. 2018 | | -0.89 [-5.32; 3.54] | |  |  |  |  |  |  |
|  |  | Clifford et al. 2018 | | -2.79 [-5.35; -0.22] | |  |  |  |  |  |  |
|  | 20-m sprint basal  24 h | Ascensão et al. 2011 | | 0.20 [0.01; 0.40] | | 0,09 [-0.02; 0.22] | | 0.1143 | 0.48 [-0.05; 1.01] | | |
|  |  | Marqués-Jiménez (a) et al. 2018 | | 0.06 [-0.02; 0.14] | |  |  |  |  |  |  |
|  | 20-m sprint basal  48 h | Ascensão et al. 2011 | | 0.28 [-0.09; 0.40] | | 0.12 [-0.17; 0.42] | | 0.413 | 0.32 [-0.87; 1.52] | | |
|  |  | Marqués-Jiménez (a) et al. 2018 | | -0.02 [-0.09; 0.05] | |  |  |  |  |  |  |
|  | MVC  24 h | Ascensão et al. 2011 | | -349.33 [-351.60; -175.36] | | -236.50 [-467.28; -5.73] | | 0.0446 | -0.70 [-1.20; -0.19] | | |
|  |  | Clifford et al. 2018 | | -113.63 [-259.63; 32.37] | |  |  |  |  |  |  |
|  | MVC  48 h | Ascensão et al. 2011 | | -265.48 [-375.30; -1558.65] | | -193.30 [-345.19; -41.40] | | 0.012 | -1.34 [-2.02; -0.66] | | |
|  |  | Clifford et al. 2018 | | -110.09 [-246.64; 26.46] | |  |  |  |  |  |  |
| **Secondary outcomes** | QS DOMS Post-match  24 h | | Ascensão et al. 2011 | | 2.00 [0.91; 3.09] | 2.02 [1.02; 3.01] | <0.0001 | | | 1.29 [0.72; 1.86] |  |
|  |  |  | Marqués-Jiménez (b) et al. 2018 | | 2.10 [-0.39; 4.59] |  |  |  |  |  |  |
|  | QS DOMS Post-match  48 h | | Ascensão et al. 2011 | | 0.64 [-0.39; 1.66] | 0.52 [-0.44; 1.48] | 0.286 | | | 0.98 [-0.40; 2.36] |  |
|  |  |  | Marqués-Jiménez (b) et al. 2018 | | -0.3 [-3.01; 2.40] |  |  |  |  |  |  |
|  | HS DOMS Post-match  24 h | | Ascensão et al. 2011 | | 1.98 [0.63; 3.33] | 1.35 [-0.61; 3.32] | 0.1786 | | | 0,54 [-1.38; 2.46] |  |
|  |  |  | Marqués-Jiménez (b) et al. 2018 | | -0.25 [-3.43; 2.93] |  |  |  |  |  |  |
|  | HS DOMS Post-match  48 h | | Ascensão et al. 2011 | | 0.56 [-0.55; 1.67] | 0.18 [-1.28; 1.64] | 0.8061 | | | 1.26 [-0.18; 2.66] |  |
|  |  |  | Marqués-Jiménez (b) et al. 2018 | | -1.30 [-4.35; 1.72] |  |  |  |  |  |  |
|  | CS DOMS Post-match  24 h | | Ascensão et al. 2011 | | 1.29 [0.20; 2.37] | 1.25 [0.20; 2.30] | 0.0188 | | | 1.20 [0.01; 2.40] |  |
|  |  |  | Marqués-Jiménez (b) et al. 2018 | | 0.80 [-2.88; 4.48] |  |  |  |  |  |  |
|  | CS DOMS Post-match  48 h | | Ascensão et al. 2011 | | -2.60 [-3.72; -1.49] | -2.23 [-3.88; -0.57] | 0.0082 | | | -0.40 [-0.52; -0.28] |  |
|  |  |  | Marqués-Jiménez (b) et al. 2018 | | -0.35 [-4.13; 3.43] |  |  |  |  |  |  |
|  | CK  24 h^a^ | | Ascensão et al. 2011 | | 754.00 [718.03; 789.96] | 753.54 [717.63; 789.46] | <0.0001 | | | 1.91 [1.12; 2.70] |  |
|  |  |  | Fullagar et al. 2016 | | 607.00 [-41.80; 1255.80] |  |  |  |  |  |  |
|  | CK  48 h^a^ | | Ascensão et al. 2011 | | 750.00 [717.74; 782.25] | 665.90 [345.43; 986.37] | <0.0001 | | | 2.12 [0.55; 3.70] |  |
|  |  |  | Fullagar et al. 2016 | | 348.00 [-253.74; 949.74] |  |  |  |  |  |  |
|  | CRP  24 h^a^ | | Ascensão et al. 2011 | | 1.65 [1.34; 1.95] | 1.62 [1.32; 1.91] | <0.0001 | | | 2.26 [1.47; 3.07] |  |
|  |  |  | Fullagar et al. 2016 | | 1.2 [0.09; 2.31] |  |  |  |  |  |  |
|  | CRP  48 h^a^ | | Ascensão et al. 2011 | | 0.15 [-0.16; 0.46] | 0.27 [-0.42; 0,97] | 0.442 | | | 0.47 [-0.05; 1.01] |  |
|  |  |  | Fullagar et al. 2016 | | 1.3 [-0.72; 3.32] |  |  |  |  |  |  |
